# Influence of virtual reality visual feedback on the illusion of movement induced by tendon vibration of wrist in healthy participants

**DOI:** 10.1371/journal.pone.0242416

**Published:** 2020-11-20

**Authors:** Salomé Le Franc, Mathis Fleury, Mélanie Cogne, Simon Butet, Christian Barillot, Anatole Lecuyer, Isabelle Bonan

**Affiliations:** 1 Rehabilitation Medicine Unit, University Hospital of Rennes, Rennes, France; 2 Inria, Rennes, France; 3 Empenn Unity U1228, Inserm, Inria, University of Rennes, Irisa, Umr Cnrs 6074, Rennes, France; University of Ottawa, CANADA

## Abstract

**Introduction:**

Illusion of movement induced by tendon vibration is an effective approach for motor and sensory rehabilitation in case of neurological impairments. The aim of our study was to investigate which modality of visual feedback in Virtual Reality (VR) associated with tendon vibration of the wrist could induce the best illusion of movement.

**Methods:**

We included 30 healthy participants in the experiment. Tendon vibration inducing illusion of movement (wrist extension, 100Hz) was applied on their wrist during 3 VR visual conditions (10 times each): a moving virtual hand corresponding to the movement that the participants could feel during the tendon vibration (Moving condition), a static virtual hand (Static condition), or no virtual hand at all (Hidden condition). After each trial, the participants had to quantify the intensity of the illusory movement on a Likert scale, the subjective degree of extension of their wrist and afterwards they answered a questionnaire.

**Results:**

There was a significant difference between the 3 visual feedback conditions concerning the Likert scale ranking and the degree of wrist’s extension (p<0.001). The Moving condition induced a higher intensity of illusion of movement and a higher sensation of wrist’s extension than the Hidden condition (p<0.001 and p<0.001 respectively) than that of the Static condition (p<0.001 and p<0.001 respectively). The Hidden condition also induced a higher intensity of illusion of movement and a higher sensation of wrist’s extension than the Static condition (p<0.01 and p<0.01 respectively). The preferred condition to facilitate movement’s illusion was the Moving condition (63.3%).

**Conclusions:**

This study demonstrated the importance of carefully selecting a visual feedback to improve the illusion of movement induced by tendon vibration, and the increase of illusion by adding VR visual cues congruent to the illusion of movement. Further work will consist in testing the same hypothesis with stroke patients.

## Introduction

Vibratory stimulation is used in various medical applications such as pain management [1] or proprioceptive rehabilitation after stroke [2]. Vibratory stimulation has a powerful proprioceptive role [3, 4] and when applied under strict conditions (frequency of 80–100 Hz, tendon target) [5], it can create illusions of movement also named kinesthetic illusions [6] (or tendon vibration inducing illusion) by apparently stimulating the brain motor areas. The neuronal activity in the primary motor area seems to be associated with the sensation of limb movement during tendon vibration [7]. There is a wide range of haptic stimulations, such as standard vibration, tendon vibration as described here, and pressure stimulation. Each tool has different influences on the sensory stimulation and the brain activity it triggers. In the literature concerning tendon vibration, we know that this kind of vibration could correspond to passive movements in term of cortical excitability in sensorimotor areas [8]. Vibratory sensation applied to a tendon, triggers the activation of local mechanoreceptors, which induces a visible elongation of this tendon. This phenomenon elicits a kinesthetic illusion antagonistic to the vibrated tendon and leads to a higher cortical activity in sensorimotor areas and a reinforcement of activation in the propriomotor loop [9–11]. The main advantage of this propriomotor loop is to be more effective than the visuomotor one, which is slower and less automatic in terms of neuronal activation [12]. It could be helpful for motor rehabilitation of neurological impairments where attention, cognitive and visual disorders can disturb the rehabilitation program.

In the last twenty years, a growing number of studies have taken interest in developing Virtual Reality (VR) tools in various fields, such as social experiment [13], haptic studies [14] and rehabilitation [15–17]. Virtual reality immersion is the perception of being physically present in a non-physical world. The perception is created by surrounding the user with images, sounds or other stimuli that provide an immersive environment. Burdea defined VR as an environment that gathers interactivity, embodiment and imagination [18]. The user can interact with a virtual environment that looks realistic enough to allow a greater feeling of immersion [19]. In this immersive state, the participant is no longer aware of their own physical state. The more immersive the virtual environment is, the more the subject adheres to it. Immersion has a special effect called "embodiment”: the subject feels present in the virtual world and interacts with it as if it was real. Embodiment depends on the quality of certain factors such as appearance or point of view in the VR environment [20]. Some studies suggested that the detailed appearance of the body contributed to the construction of the body image in VR [21]. Kim also demonstrated the importance of the correspondence between the properties of the real human body and the adaptation made in VR to obtain the best possible incarnation and therefore the best illusion [22]. The role of embodiment in VR seems valuable to immerse participants in a controlled environment and create kinesthetic illusions [23]. Combining VR interface with haptic devices tends to increase the feeling of embodiment described in the literature, by giving a congruent tactile feedback to a visual immersive environment [24]. Rinderknecht also proved that the addition of the virtual reality enhanced the perception of the illusory movement induced by tendon vibration in healthy participants [25].

The aim of the present study is to determine the optimal parameters that can enhance an illusion of movement obtained by using a tendon vibration and VR. If the technical parameters of the vibration are already determined [25–28], the visual conditions facilitating and enhancing the creation of a kinesthetic illusion are not yet known. Some studies tend to show that the kinesthetic illusion is more important when the participant receives a visual cue congruent with the kinesthetic illusion induced by the vibration [29–31] compared to the case of a lack of vision of the concerned target during the vibration period [32–34]. These studies focus on healthy participants with protocols using mainly the “illusion mirror paradigm" [35] without using Virtual Reality tools during tendon vibration. On the other hand, Caola showed that the illusions of ownership and movement were higher when concomitant synchronous visuo-tactile stimulations were provided using VR immersion [36]. Other studies have also demonstrated the interest of using a virtual environment congruent with the movement of our limbs to allow a better illusion and feeling of embodiment [37], or the combination of visuomotor and visuotactile stimulation on the virtual body ownership illusion [38]. Concerning the rubber hand illusion protocol [39], the illusion decreased when the tools were not congruent with their visual appearance and expected tactile consequences [40].

Our research hypothesis is that a virtual visual condition congruent to the illusion of movement produced by a tendon vibration enhances the intensity of illusion rather than the lack of visual cue or an incongruent cue. Therefore, we tested in healthy participants 3 virtual visual conditions associated with wrist tendon vibration: a moving virtual hand, a static virtual hand and no virtual cue and evaluated the illusion of movement felt in each condition.

## Materials and methods

### Study design

**Fig 1:**
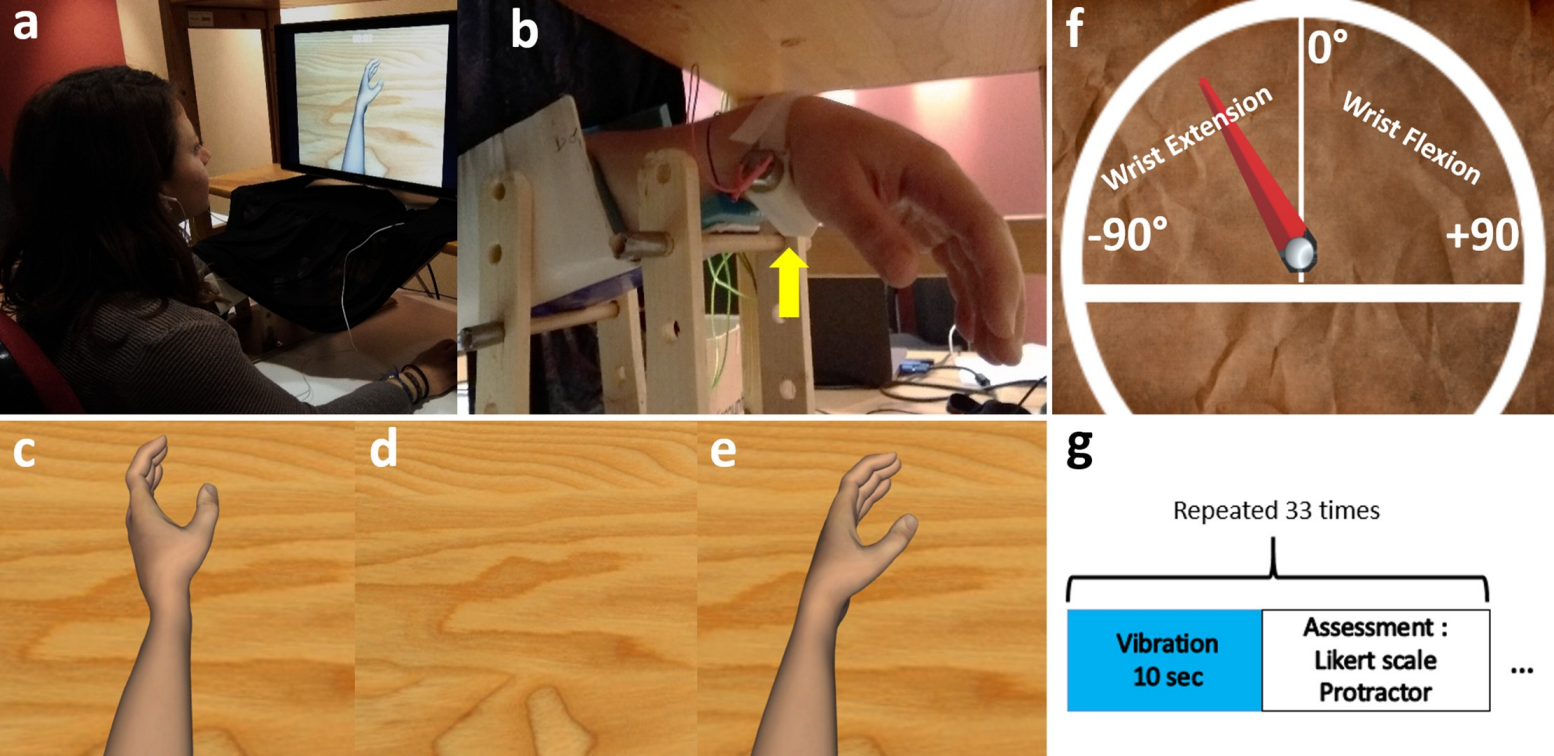
Apparatus used in the experiment (exemple for a right-handed participant). **a-b)** Set-up of the vibrator. A black curtain covered the forearm of the participant. **c-d-e)** Visualization of the three virtual visual conditions (respectively Moving, Hidden, Static condition). A black arrow (not visible during the experiment) indicates the movement of the wrist in the Moving condition, from flexion to extension. **f)** Measure of sensation of displacement with the protractor. « -90° » indicates an extreme wrist extension in the case of a left upper limb. The notes «values of degree» and « wrist extension, wrist flexion » are not visible by the participant during the experiment.

We conducted in 2019 a monocentric randomized controlled pilot study in the Rehabilitation Unit of Rennes University Hospital in France. The study was promoted by the Rennes University Hospital Center and obtained the approval of the Ethics Committee of Strasbourg University, France, on October 8th, 2019 (record number: 19/62-SI 19.07.05.46737). An information letter was provided to the participants including: the aims of the study, the protocol, the involved risks and insurance notifications. Written consent was obtained from each participant prior to testing. This study has been recorded in Clinical Trials under the following record number NCT04130711. No changes to the study design were made after approval by the ethics committee. The participant in the picture (Fig 1A) in this manuscript has given written informed consent (as outlined in PLOS consent form) to publish these case details.

### Participants

Volunteer healthy participants were recruited using a public information in the Department of Rehabilitation unit of Rennes University Hospital and of the Medicine Department of Rennes University. A total of 30 healthy participants (Mean± Standard Deviation): 24.93±3.79 years old, Min = 21, Max = 35 participated to the study, with 22 men (73,33%) and 8 women (26,66%). All healthy participants fulfilled the following inclusion criteria: age between 18 and 80 years old; no previous history of neurological illness. We asked the participants if they had a neurological history such as brain injury, brain surgery, epilepsy. No specific questionnaire was used. Participants deprived of freedom and with a legal incapacity were excluded from the study. Concerning the number of participants, a cohort of 30 participants was sufficient to obtain a good statistical power when using a within design. Furthermore, the corresponding literature motivating the research hypothesis includes studies involving about 11 [29], 13 [31], 3 [33] or 8 participants [34].

## Experimental procedure

### Procedure

Participants sat in a typical office chair with armrest and adjustment of seat height and backrest inclination in front of a computer screen. Their non-dominant arm was positioned in a shell to keep it on the edge (Fig 1A and 1B), with the hand hidden from view by a black cloth, without support at the top (Fig 1A). Laterality was determined by an Edinburgh questionnaire. A vibrator set on their flexor carpi tendon. Tendon vibration was applied during 10 seconds consecutively at the frequency of 100 Hz in order to induce an illusory movement. For each tendon vibration trial, participants saw random visual conditions on the computer screen in front of them: a virtual hand moving in the same direction as the wrist extension (Moving condition) (corresponding to the movement that the participant could feel if the tendon vibration induced a correct illusory movement); no hand at all with an empty screen (Hidden condition); a static virtual hand (Static condition) (Fig 1C, 1D and 1E). Immediately, after each trial the participants were asked to indicate on a virtual protractor the maximal angle to which the illusion of wrist movement had gone (Fig 1F) and how much they felt the movement illusion by using a Likert scale [41] from 1 to 7 (with 1 = no illusion at all; 4 = moderate intensity of illusion of movement; 7 = strong intensity of illusion of movement). For each participant, 33 trials were conducted i.e. 11 trials per visual condition (Fig 1G). The first three trials were not included in the analysis, and were useful to check on 3 consecutive vibrations if the vibrator was well positioned, and if the evaluation modalities were understood by the participant. At the end of the experiment, participants filled out a questionnaire to determine if the participants had already tried such vibrating devices, get subjective data on vibration comfort, get the preferred visual condition, understand if the intensity of illusion was sufficiently felt. First, we explained orally that the subject would receive bursts of vibration to their wrist, which might give them a feeling of movement. We did not specify which movement it might be (hand, finger…) nor in which direction it would occur. Then we explained to them that during these vibrations, they would see on a screen a virtual hand resembling theirs, which might move or not. Again, we did not describe the direction of the hand's movement on the screen. Then we gave our instructions in writing the same way. We systematically reminded the subjects to concentrate well on their sensations. In this way, we avoided influencing participants on potential outcomes or biased information. We checked that they understood the instructions, and we checked the consistency of the answers on the protractor and the Likert scale on the first trial to be sure that they had understood the guidelines.

### Visual feedback

**Fig 2:**
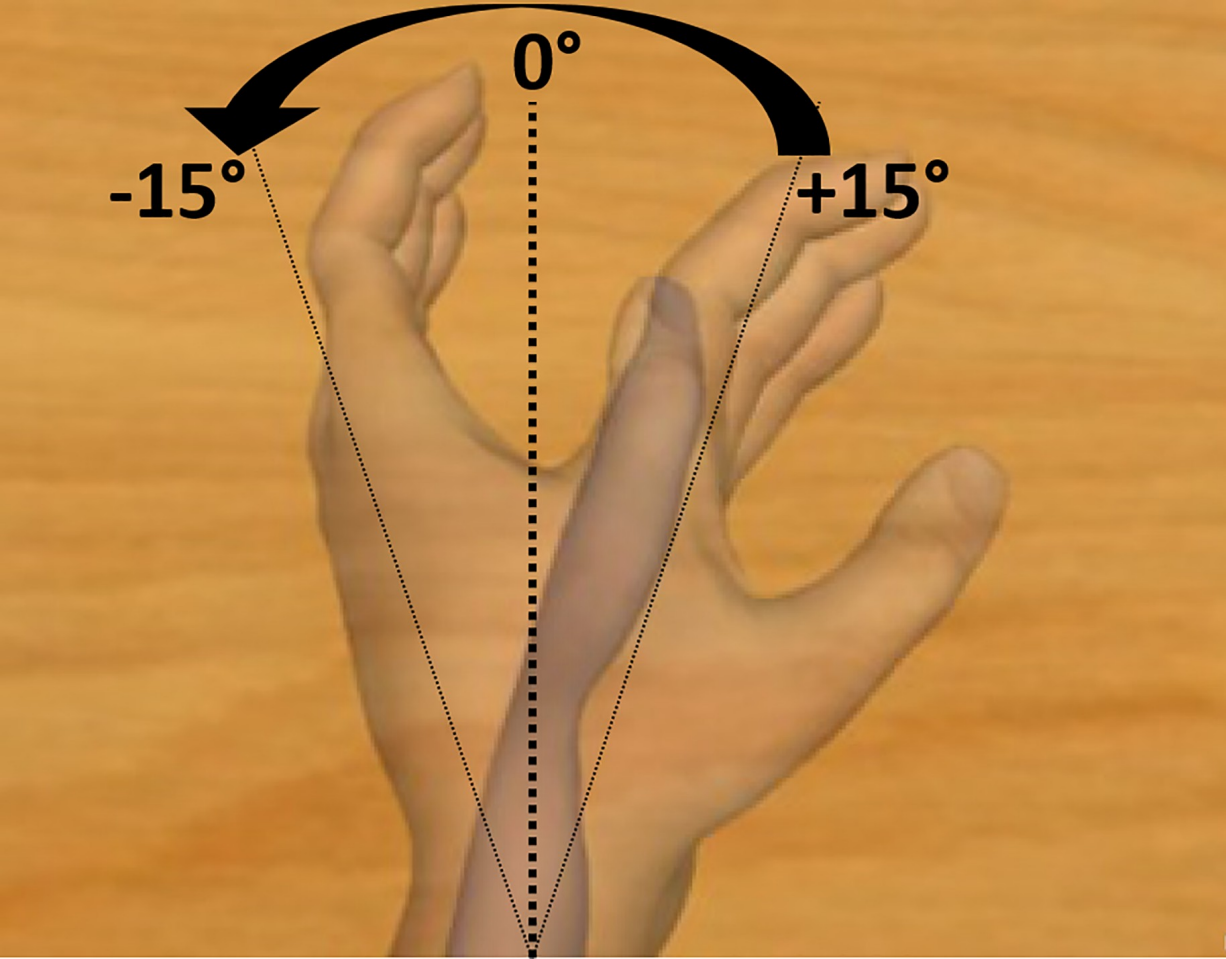
Description of the moving condition. Movement from wrist flexion to wrist extension, with a total displacement of 30 degrees around the rest position. The values and arrow are not visible by the participant during the experiment.

Visual feedback of the performed mental task was given to the participants by using Unity software, and the virtual scene was composed of a homemade neutral and white skin upper limb avatar. The scene was displayed on a 17 inch-LCD monitor, was rendered from the point of view of the virtual avatar and the monitor was positioned in order to match participant’s first perspective. The movement executed by the virtual hand was either: 1) Moving condition (Figs 1C and 2): an extension of the non-dominant wrist with a total displacement of 30 degrees from the resting position, at speed of 3 degrees per second, congruent with the illusory movement which was expected by the application of a flexor carpi tendon vibration [8], or 2) Hidden condition (Fig 1D): an empty surface corresponding to the space occupied by the virtual hand or 3) Static condition (Fig 1E): a static virtual hand of the non-dominant wrist in front of the participant. The visual clue was available to represent a left or right hand depending on the laterality of the participant.

### Vibratory device

**Fig 3:**
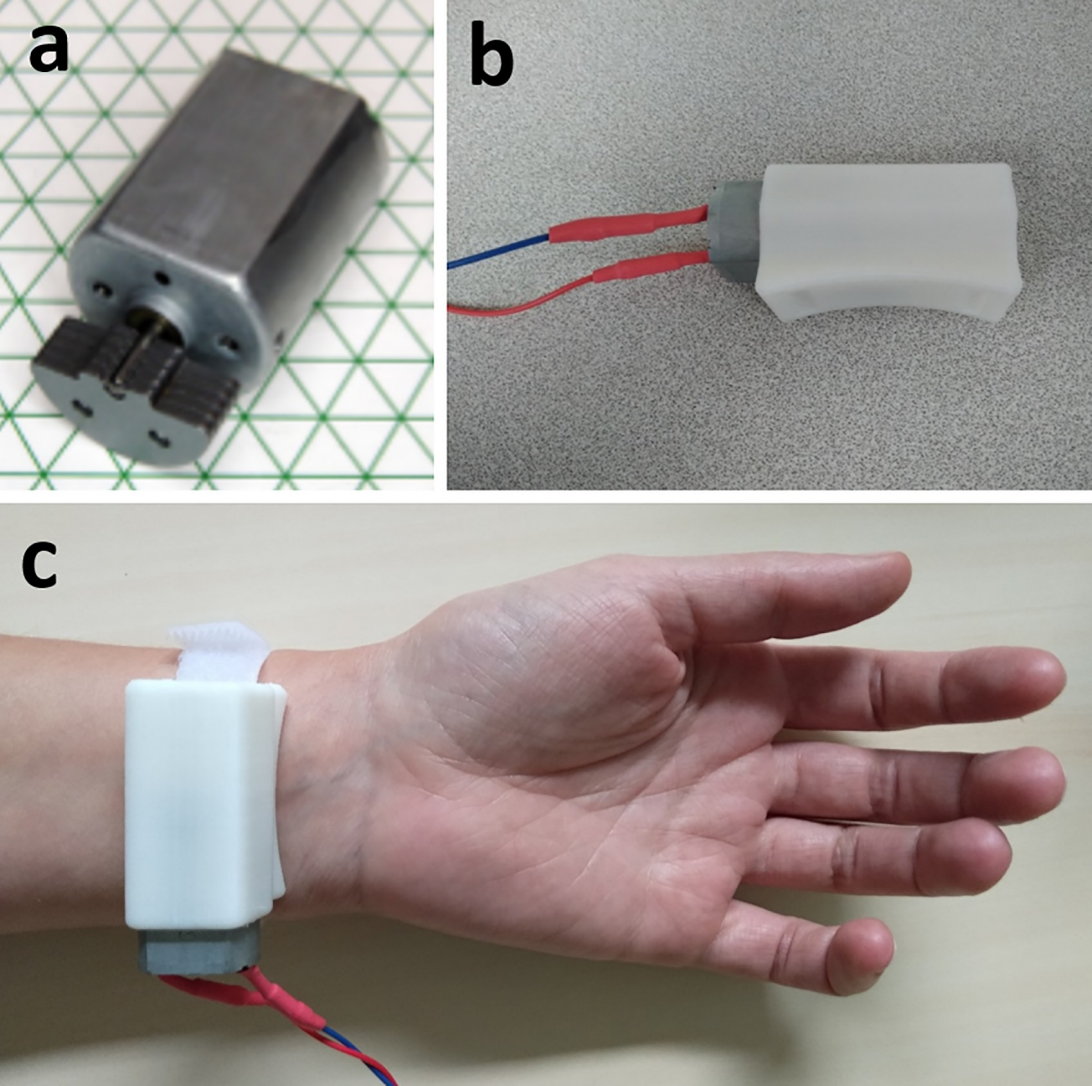
Pictures of the vibratory device UniVibe™. **a)** Raw vibration motor. **b)** Vibration motor device linked to the Arduino® and inside a sound box. **c)** Wrist placement.

The device used in this work is a UniVibe™ Model 320–105 vibratory unit (Fig 3), which was composed of an actuator with an adjustable position and orientation that can be finely positioned on flexor carpi tendon and maintained on skin with hook-and-loop fastener. We created a sound box by 3D print to protect the skin from the motor and allow a better sensation of vibration. An Arduino® controls the vibration motor. Actuation was obtained by using a linear voice-coil, allowing to accurately modulating the frequency patterns required for eliciting the motor illusion. The vibration frequency was determined by the rotation of the mass. The diameter of the skin tactor was 25 mm. In this study, we applied a frequency of 100 Hz, an amplitude of 5G, and voltage of 3.3 V based on the literature [5, 26, 27] to elicit movement illusion.

### Collected data

Primary outcome measure was the angle of motion felt in degrees during each vibration. The participants used a computer mouse (with their free hand) which allowed them to move the needle of the protractor. The participants could steer the needle from -90° (wrist in extension) to +90° (wrist in flexion) with all possible shades of degrees. They noted the direction of illusion of movement they felt on a protractor (Fig 1F). An angle of 0° meant no illusion at all (resting position), while negative degrees meant a sensation of wrist’s extension up to -90° and positive degrees meant a sensation of wrist’s flexion up to +90°. The protractor was available for right-handed or left-handed subjects, with negative degree values describing a wrist extension. On the screen, the virtual hand moved at an angle of 30° from a discreet wrist flexion to a discreet extension (Fig 2). Secondary outcome measures were the intensity of illusion of movement noted on the Likert scale [41] (with 1 = no illusion at all; 4 = moderate intensity of illusion of movement; 7 = strong intensity of illusion of movement) after each vibration and the preferred visual condition. Data was collected in Data Archiving and Networked Services (DANS) database.

### Statistical analysis

A descriptive analysis of all variables used in this study were performed. Qualitative variables were described with frequencies and their related percentages. Quantitative variables were divided into two groups:

1. Variables following the normal distribution using the mean ± standard deviation
2. Variables not following the normal distribution using the median and interquartile intervals.

Statistical tests were performed with SPSS Version 22 and R Version 3.6.2 softwares. The repeated measures analysis of variance (ANOVA) has revealed a violation of the assumption of sphericity according to Mauchly's test [42] in particular for one of the main judgment criteria (*i*.*e*., the difference in motion illusion in degrees): χ^2^ = 113.40, p<0.001. Thus, a non-parametric approach was followed. A within-group analysis comparing the 3 visual virtual conditions (static condition, moving condition, hidden condition) was conducted using Friedman tests and then 2 by 2 conditions using post-hoc tests (Wilcoxon signed rank test) corrected with Bonferroni.

## Results

**Fig 4:**
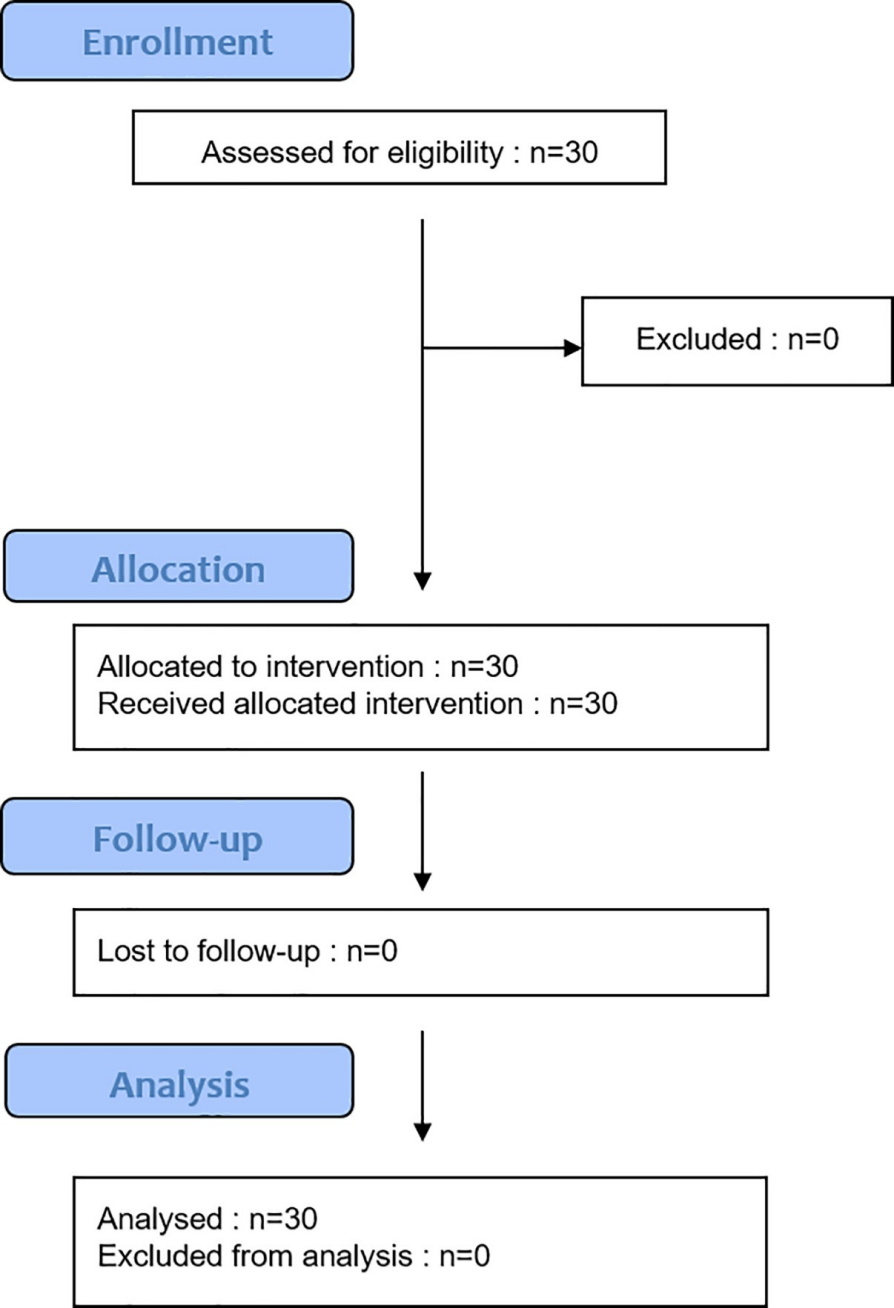
Flowchart of the experiment.

This is the flowchart of the experiment (Fig 4).

### Sensation of wrist’s extension

**Fig 5:**
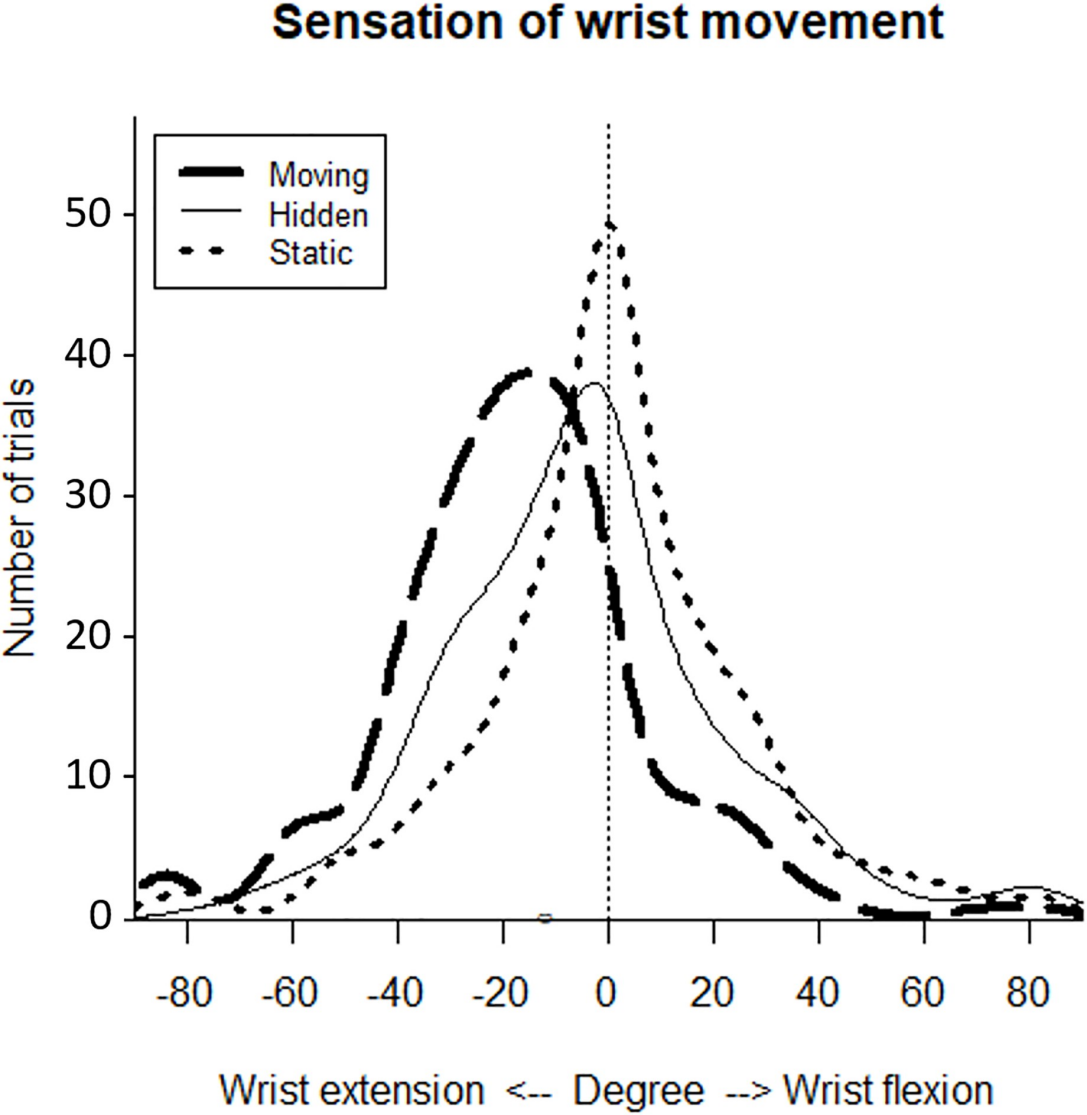
Frequency of sensation of wrist extension. Smoothed histogram of the frequency of sensation of wrist displacement in each condition averaged in healthy controls. The vertical line represents the zero degree axis.

The mean (±SD) sensation of wrist’s extension was respectively -17.59 (±24.77) for the Moving condition, -4.14 (±27.31) for the Hidden condition and 0.44 (±26.23) for the Static condition (Fig 5). Comparison of the repeated measures was performed using Friedman’s test showing a statistically significant difference in the conditions, χ^2^ = 113.40, p<0.001). Post-hoc analysis with a Bonferroni correction applied, showed that the Moving condition induced a higher sensation of wrist’s extension than the Hidden condition and the Static condition (p<0.001). The Hidden condition also induced a higher sensation of wrist’s extension than the Static condition (p<0.01). We then compared results between women and men. There were 8 women and 22 men. We did not find any significant results between the groups in each condition (Kruskal Wallis test), respectively for the Moving condition (H = 1.35, p = 0.24), the Static condition (H = 0.45, p = 0.50), the Hidden condition (H = 0.51, p = 0.48).

### Intensity of the illusion of movement

**Fig 6:**
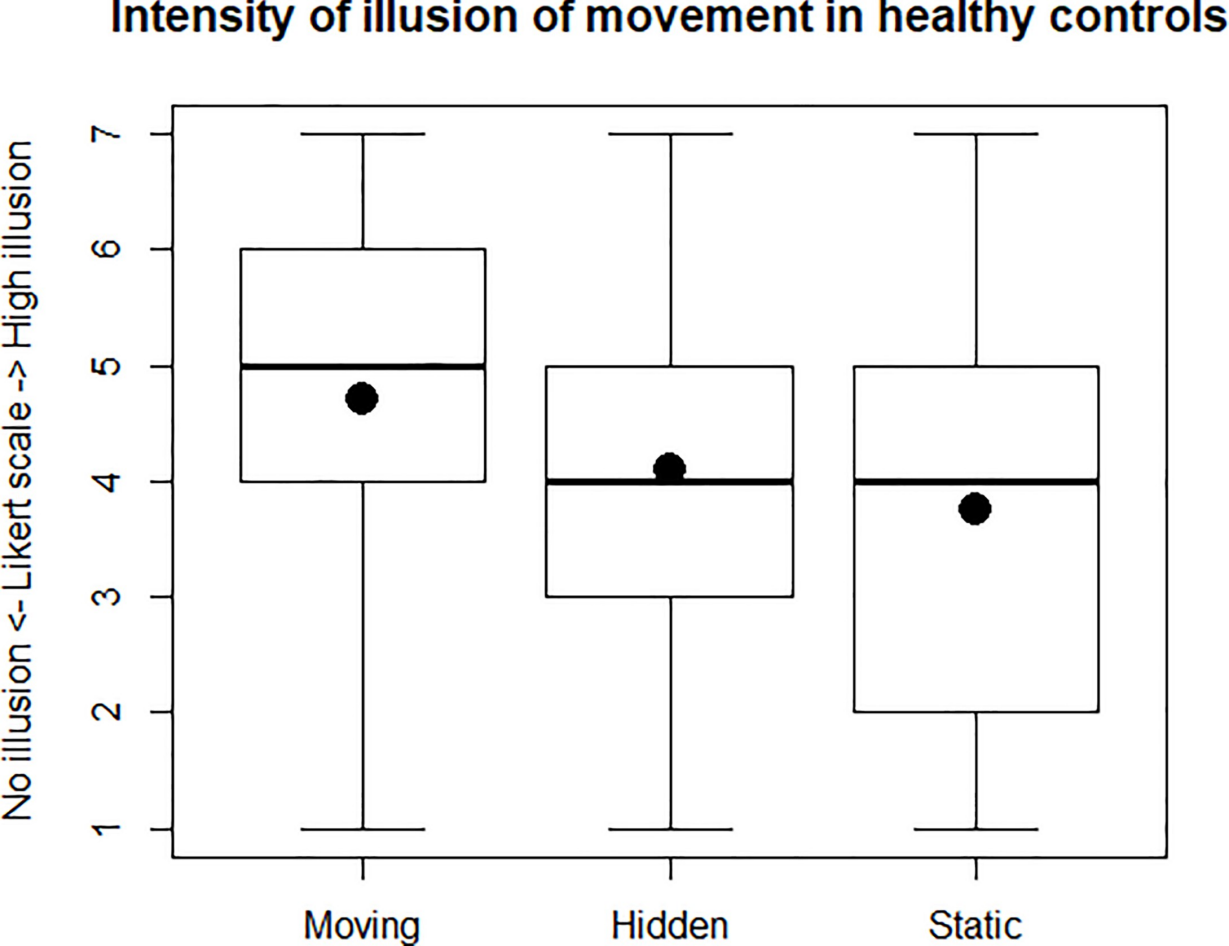
Intensity of illusion of movement. Boxplot about intensity of illusion of movement felt for each condition, averaged in all healthy controls (respectively for Moving, Hidden, Static condition). Likert scale from 1 to 7: 1 means “no illusion”, 7 mean “very high intensity of illusion”. The dots represent the means.

The mean (±SD) Likert ranking was respectively 4.70 (±1.56) for the Moving condition, 4.11 (±1.64) for the Hidden condition and 3.76 (±1.70) for the Static condition (Fig 6). Comparison of the repeated measures was performed using Friedman’s test showing a statistically significant difference between the 3 visual conditions concerning the Likert scale ranking (χ^2^ = 67.93, p<0.001). Post-hoc analysis with a Bonferroni correction applied, showed that the Moving condition induced a higher intensity of illusion of movement than the Hidden condition (p<0.001) and the Static condition (p<0.001). The Hidden condition induced a higher intensity of illusion of movement than the Static condition (p<0.01).

### Subjective reports of participants

Among our 30 participants, 27 were right-handed (90%) and 3 were left-handed (10%). Four participants had already had a small experience of illusion of movement induced by tendon vibration (13.3%). The participants’ preferred condition to facilitate the illusion of movement was the Moving one (Number of participants (n = 19, 63.33%), then the Hidden condition (n = 6, 20%), then the Static condition (n = 2, 6.67%), and some participants did not prefer any of the 3 conditions (n = 3, 10%). The type of illusion felt by the participants was mainly a wrist extension (n = 16, 53.33%), then a wrist flexion (n = 7, 23.33%), then a wrist supination (n = 6, 20%) and a fingers extension (n = 1, 3.33%). Most participants felt the illusion of movement easily while they experienced the Moving and Hidden conditions compared to the Static one (respectively 83.3% (n = 25), 66.7% (n = 20), and 40% (n = 12)). When participants felt an illusion of movement, it rather appeared at the onset of the vibration period in the Moving condition (n = 15, 50%), or at the middle of the vibration period in the Hidden and Static conditions (respectively n = 13, 43.33% and n = 12, 40%). The illusion of movement lasted approximatively 10 seconds for the participants in the Moving condition (n = 12, 40%) whereas it lasted less than 5 second in the Hidden and Static condition (respectively n = 12, 40% and n = 14, 46.66%). During the experiment, some participants (n = 3, 10%) experienced a transient uncomfortable feeling of paresthesia or itching on their wrist and/or their hand, without any need to stop the experiment.

## Discussion

This study investigated the contribution of virtual visual cues to improve the illusion of movement induced by wrist tendon vibration in healthy participants. The results confirmed our main hypothesis that the illusion of movement was higher when the movement of the virtual hand seen on the screen was congruent to the sensation of illusion felt. The Moving condition was significantly superior to the Hidden and Static condition in terms of sensation of wrist extension (Fig 5), intensity of illusion (Fig 6) and comfort for the participants.

The Hidden condition was also superior to the Static condition in terms of sensation of wrist displacement and intensity of illusion of movement which was expected regarding the existing literature [33, 34, 43]. These results could be explained by the incongruent visual cue given to the participant and disturbing the production of illusory movements. The participants also indicated that the illusion of movement rather started after few seconds and lasted about 5 to 10 seconds when the illusion was good. The onset of the illusion matched with the data found in the literature [27], i.e. approximately 5 seconds. The best duration of the illusion in the literature seemed to be between 10 and 60 seconds [27, 28, 44], which is also consistent with our data.

Surprisingly, we found that the illusion of movement that what felt did not only consist in a wrist extension in all participants, contrary to what is described in the literature [5, 34] when a vibration on the flexor carpi tendon is applied. Indeed, in our experiment, only 4 participants felt exclusively a wrist extension during the entire vibration period. All other participants felt a wrist extension, but also a wrist flexion, even a wrist supination sometimes, without any movement of the participant or the vibratory during the experiment. All these sensations seemed random, not depending on a specific visual condition, except for the Moving condition which more frequently induced a sensation of wrist extension. In this virtual Moving condition, the illusion of movement seemed lower when the participants felt another illusion that wrist extension, because the illusion became incongruent to the visual cue, regarding to the reports of the participant. Nevertheless, even though the goal of tendon vibration was a wrist extension in this study, the illusion of movement was well present in all participants in the Moving condition, and could also be effective in stimulating brain motor areas [8]. In this protocol, the combination of the kinesthetic illusion and the visual index of a corresponding virtual hand can be correlated with action-observation (AO) studies known to stimulate sensori-motor areas [45, 46].

The interest of stimulating the wrist extension was to be close with the aim of motor rehabilitation in stroke patients who suffer from motor control deficiency and often spasticity in their upper limb extremity [47]. It could lead to a closed fist, and one main goal of the rehabilitation care is to open the hand and stretch the wrist in order to avoid vicious deformations [48, 49]. We tested the participants with their non-dominant upper limb. First, we found in the literature that the illusion of movement induced by tendon vibration could be higher on the non-dominant limb [27]. Then, the aim was to match a healthy population with a chronic stroke population. The post-stroke subjects often needed to re-lateralize themselves to use the non-injured upper limb in case of incomplete recovery.

However, our experiment presented some methodological limitations. Above all, the between-design was not applicable here. Each participant had 33 vibration tests (10 in each condition). All the tests were performed in a randomized order, so that the first vibration tests could be randomly any of the 3 conditions repeated several times in a row. Moreover, as explained below, to feel an illusion of movement induced by tendon vibration, the participants needed to be completely relaxed. Subjects at the beginning of the study tended to not be relaxed due to experiencing new vibrations. It also took them some time to adjust to the virtual world and to immerse themselves into the VR tool. Sometimes, they became distracted by the investigator due to repositioning the vibrator, particularly during the first tests. They also needed some time to understand the measurement system with the Likert scale and the protractor. For all these reasons, it was necessary to remove these initial tests from the main analysis and therefore not do a between-design analysis.

One explanation concerning the unexpected sensation of wrist flexion and supination during tendon vibration could be the complexity of the wrist anatomy. Flexor carpi tendon are numerous in a very little area in the wrist and some of them have several functions as flexion but also supination. The vibratory device used in this experiment conformed with the device found in the literature, with a size including the width of the wrist. This type of device cannot be precise to the point of targeting a single tendon. Further studies would be necessary to develop more precise vibratory devices. Then, during the experiment some participants tended to tense up when they received the vibration, and could develop some Tonic Vibration Reflex (TVR) events inducing flexion illusions [32].

Stroke is the main cause of severe acquired disability in adults in developed countries [50, 51]. It can lead to a severe and persistent upper limb motor damage without recovery of a useful grip in up to 60% of stroke subjects [52]. Because it strongly impacts stroke subjects’ daily life autonomy and independence, motor function recovery of the upper limb is a major rehabilitation goal [53].

Vibration therapies seem interested in this field as demonstrated by the large number of studies on the literature [10, 54, 55]. In this context, tendon-vibration inducing illusion of movement appears as an attractive tool in this population to improve cortical excitability, neural plasticity and motor function [56], even if the injured people have no motor function at the onset of the rehabilitation.

Next steps of the study will be to test the same hypothesis with stroke patients to quantify if and how an illusion of movement could be obtained in the same conditions. Chancel et al. [57] tested in elderly the ability to perceive self-hand movements based on multisensory feedback with vibration on thumb. Results showed that the illusion of movement induced by a tendon vibration was slower and weaker to appear in elderly people that in a younger population. Stroke patients are mainly older than our study population [50], thus we can expect a weaker illusion of movement felt with stroke patients. In addition, stroke people often suffer from others symptoms such as attention disorder which can decrease the ability to focus or hypoesthesia. Sensory inputs and sensorimotor integration can be disrupted. The current literature [58, 59] remains unclear about the effectiveness of central integration of peripheral vibration in this population.

In conclusion, our results showed that virtual visual cues congruent to the illusion of expected movement enhanced the illusion of movement induced by tendon vibration. Our study is the first which demonstrates the benefits to use VR cues with tendon vibration to improve the illusion of movement in healthy participants. Moreover, it confirms that congruent visual cues are greater than hidden objects or even static visual cues.

Our results pave the way to the design of new tools in rehabilitation for stroke patients using Virtual Reality associated with haptic stimulation to improve motor recovery in post-stroke subjects.

## Supporting information

S1 File (XLSX)

S2 File (XLSX)

S3 File (DOCX)
